# Patient’s view on: decreasing cardiovascular events after kidney transplantation: a Finnish analysis covering 1990 to 2019

**DOI:** 10.1093/ckj/sfag223

**Published:** 2026-07-03

**Authors:** Brooke Huuskes

**Affiliations:** Centre for Cardiovascular Biology and Disease Research, Department of Microbiology, Anatomy, Physiology and Pharmacology, La Trobe University, Bundoora, Australia

A cardiovascular event, infection and cancer are the leading causes of mortality in the kidney transplant population. As a transplant recipient, this is very alarming and something I think about a lot. As someone who was transplanted at a young age, it was very confronting knowing that I have a significantly higher risk of having or dying from a cardiovascular event. A heart attack was something that happened to old people, not someone my age.

The Finnish study demonstrates that cardiovascular events have decreased in kidney transplant patients since the 1990s. This is a welcome sigh of relief for patients! In fact, the authors show that non-fatal cardiovascular events (acute myocardial infarction and stroke) have decreased more in the kidney transplant population than in the general population. This suggests that the methods for screening and preventing cardiovascular disease which led to this decrease in the general population are not the only reason for the observed decrease in the transplant population. Rather, transplant era is likely a contributing factor, with advances in immunosuppressive agents leading to better graft survival and hence, cardio protection.

These findings are extremely encouraging and suggest that advances in the medication regimes of kidney transplant recipients can drastically improve outcomes. I am really interested in the outcomes of clinical trials focusing on the use of sodium-glucose cotransporter 2 (SGLT2) inhibitors in kidney transplantation. Is a drug that is known for cardiac protection beneficial in transplant recipients?

The paper acknowledged some limitations, including that the data come from a single country and therefore the findings may not be applicable to other transplant populations. With a bigger dataset it would be interesting to expand on these findings to include people who have had multiple transplants, and to stratify the data by age. Specifically, looking at the pediatric population, who are more likely to have multiple transplants and periods on dialysis, we can ask: do these factors increase the risk profile of cardiovascular disease and if/how they have changed over time? But more importantly, we can ask: what interventions decrease the risk of cardiovascular disease?

As a young transplant recipient, studies like this one give me hope! Not only for my future but for the future of many transplant recipients to come.

